# Variation in general practice referral rate to acute medicine services and association with hospital admission. A retrospective observational study

**DOI:** 10.1093/fampra/cmac097

**Published:** 2022-09-05

**Authors:** Marcus J Lyall, Dan Beckett, Anna Price, Mark W J Strachan, Clare Jamieson, Catriona Morton, Drummond Begg, Johanne Simpson, Nazir Lone, Allan Cameron

**Affiliations:** Department of Medicine, Royal Infirmary of Edinburgh, 51 Little France Cres, Edinburgh EH16 4SA, United Kingdom; Department of Acute Medicine, Forth Valley Royal Hospital, Stirling Rd, Larbert FK5 4WR, United Kingdom; Department of Public Health, Medical Statistician, Western General Hospital, Crewe Rd S, Edinburgh EH4 2XU, United Kingdom; Metabolic Unit, Western General Hospital, Crewe Rd S, Edinburgh EH4 2XU, United Kingdom; Gullane Medical Practice, Hamilton Road, Gullane, East Lothian EH31 2HP, United Kingdom; Craigmillar Medical Group, 106 Niddrie Mains Road, Edinburgh EH16 4DT, United Kingdom; Penicuik Medical Practice, 37 Imrie Place, Penicuik EH26 8LF, United Kingdom; Department of Medicine, Royal Infirmary of Edinburgh, 51 Little France Cres, Edinburgh EH16 4SA, United Kingdom; Usher Institute, University of Edinburgh, Edinburgh EH8 9AG, United Kingdom; Department of Acute Medicine, Acute Assessment Unit, Jubilee Building, Glasgow Royal Infirmary, Glasgow G4 0SF, United Kingdom

**Keywords:** acute disease, emergency medicine, epidemiology, general practice, primary health care, referral and consultation

## Abstract

**Background:**

Variation in general practice (GP) referral rates to outpatient services is well described however variance in rates of referral to acute medical units is lacking.

**Objective:**

To investigate variance in GP referral rate for acute medical assessment and subsequent need for hospital admission.

**Methods:**

A retrospective cohort study of acute medical referrals from 88 GPs in Lothian, Scotland between 2017 and 2020 was performed using practice population size, age, deprivation, care home residence, and distance from hospital as explanatory variables. Patient-level analysis of demography, deprivation, comorbidity, and acuity markers was subsequently performed on referred and clinically assessed acute medical patients (*n* = 42,424) to examine how practice referral behaviour reflects clinical need for inpatient hospital care.

**Results:**

Variance in GP referral rates for acute medical assessment was high (2.53-fold variation 1st vs. 4th quartile) and incompletely explained by increasing age and deprivation (adjusted *R*^2^ 0.67, *P* < 0.001) such that significant variance remained after correction for confounders (2.15-fold). Patients from the highest referring quartile were significantly less likely to require hospital admission than those from the third, second, or lowest referring quartiles (adjusted odds ratio 1.28 [1.21–1.36, *P* < 0.001]; 1.30 [1.23–1.37, *P* < 0.001]; 1.53 [1.42–1.65, *P* < 0.001]).

**Conclusions:**

High variation in GP practice referral rate for acute medical assessment is incompletely explained by practice population socioeconomic factors and negatively associates with need for urgent inpatient care. Identifying modifiable factors influencing referral rate may provide opportunities to facilitate community-based care and reduce congestion on acute unscheduled care pathways.

**Key messages**

- The decision to refer a patient for urgent medical assessment is complex.
- Demography and practice list size incompletely explain observed variance.
- Patients from high referring practices are less likely to require inpatient care.

## Introduction

A small fraction of people seen in primary care each year are referred to hospital services for acute medical care. Acute medical assessment units are designed to provide urgent assessment and treatment for patients with potentially life-threatening conditions for whom primary care physicians (general practitioners) deem outpatient or community management impractical or unsafe.^1^ The majority of patients referred to acute medical care are appropriate and either hospital admission is arranged, urgent care given with safe discharge or serious pathology excluded. Referrals continue to increase^2,3^ and, when they exceed the capacity of an assessment unit, can negatively affect patient experience and treatment outcome.^4^

The “NHS Long Term Plan,” a UK government strategy launched in January 2019 aspires to treat patients where possible in the community and primary care setting.^5^ Similarly, the national clinical strategy for health in Scotland aims to reduce variation in practice with a much greater focus on supported self-management, anticipatory care planning, and community-based medical treatment.^6^ Historically, variation in general practice (GP) referral rates to outpatient secondary care has been widely reported, however data relating to referral rates for acute medical assessment remain sparse.^7–10^ Duffy et al. described acute medical admission rates in Tayside, Scotland, in 1996/1997 and reported a variation in emergency admission rates to hospital of 1.8-fold between the top and bottom deciles of practices. The majority of this variance (64%) was attributed to deprivation status and age of the practice population.^11^ Blatchford et al. described a similar study in Glasgow, Scotland, also examining admission rates, and reported a near 2-fold variation between top and bottom deciles after adjustment for age, sex, and deprivation score of each practice population.^12^ Both these studies addressed the question of variation in *admission rate* of the practice population rather than *referral rate* by the general practitioners for acute hospital medical assessment. Further they included patients admitted via ambulance emergency and self-attendance and as such variation in active GP referral behaviour for acute medical assessment remains unclear.

**Fig. 1.**
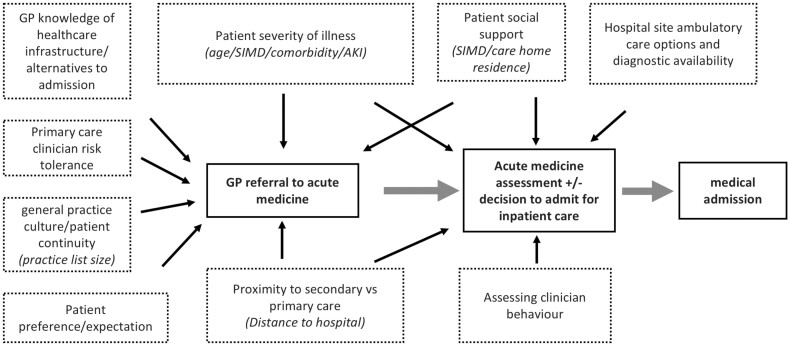
Conceptual framework around acute medicine referral from primary care. Conceptual framework of patient, practice, clinician, and healthcare infrastructure factors affecting GP referral for and subsequent admission following acute medical assessment. Measurable variables indicated in italics.

In most large UK centres, triage of individual urgent referrals to acute medicine by secondary care medical staff is not feasible and so acute medicine assessment workload is influenced substantially by clinical decision-making in primary care. While the decision to refer a patient for acute medical assessment rests with the assessing general practitioner, multiple variables feed into this decision including patient actual or perceived acuity of illness, presence of social support, patient and practitioner preference and expectation, distance from hospital, patient comorbidity, and presence of accessible alternatives to admission. Similar factors will influence the decision to admit a patient after initial hospital clinical assessment when coupled with initial examination and investigations and the preference of the assessing acute clinician (Fig. 1).

This retrospective study aimed to describe GP referral variation for acute medical assessment and to evaluate the extent to which this variation was explained by these complex socioeconomic and demographic factors and subsequently to understand how referral variation related to need for inpatient hospital care. Therefore, 2 distinct analyses were performed.

1) Individual GP-level analysis of referral rates for acute medical assessment and associations with practice population-level variables of age, deprivation, hospital proximity, care home residence, and practice list size.
2) Patients referred for acute medical assessment arrive at the receiving hospital and undergo examination and baseline laboratory and radiological examination in an assessment area. If inpatient admission is subsequently needed for ongoing clinical care then the patient is admitted. If inpatient care is not required the patient is discharged for community-based follow-up. Need for inpatient care was used as a marker of “appropriateness” of referral. Patient-level analysis was performed for this cohort including socioeconomic factors, markers of comorbidity, biochemical markers of clinical acuity, and the referral behaviour of the source practice.

## Methods

Data were obtained from the TRAKcare inpatient clinical management system (Intersystems, USA), which is in use in all hospital sites in NHS Lothian, a health board providing care for around 800,000 patients in South East Scotland. Data were examined from secondary care hospitals over a 2 year 9 month period from April 2017 to January 2020 (Royal Infirmary of Edinburgh and Western General Hospital, Edinburgh). The study period was truncated at January 2020 due to the reported changes in health seeking behaviour during the COVID-19 pandemic lockdown.^13^ There are 121 practices in NHS Lothian. Twenty-six practices admit to an alternative hospital site for which data were unavailable and were thus excluded. The university practice and homeless access practice were excluded as were 2 practices which merged during the study period and 3 practices with incomplete practice population data leaving 88 practices in total with a combined patient population of 666,112. For practice-level analysis, practice list size, percentage of practice population over 65, percentage in the 2 most deprived Scottish Index of Multiple Deprivation (SIMD) quintiles, and percentage patient population in care home residency were obtained from Public Health Scotland Information Services Division (www.isdscotland.org) and examined as continuous variables. SIMD uses 7 domains (housing, skills and training, income, employment, health, education, geographic access and crime) to produce an area-based ranking index including standardized mortality ratio, mental health prescribing data and hospital admissions for drug and alcohol use and is used as an estimate of comorbidity and deprivation.^14^ Distance from the major hospital admission site for each practice was calculated using the geographical coordinates of each practice postcode and “geosphere” package in R.^15^ To assess GP referral rates, patients referred from GPs in Lothian for acute medical assessment were calculated (referrals per 100 patient-years) for each practice. Only patients from routine in hours GP service were included (Monday to Friday 0800–1800). Referrals from other sources (emergency medicine, GP out of hours service, self-presentation) and to other specialties (e.g. surgical assessment, minor injury assessment) were excluded. For patient-level analysis individual SIMD quintile was obtained from the SIMD database and examined as an ordinal variable,^16^ Charlson score was calculated using “comorbidity” package in R based on hospital discharge codes from the preceding 5 years and examined as an ordinal variable (0, 1–2, >2) based on population frequency.^17^ Care home residence was identified from individual patient address and examined as a dichotomous variable. AKIN acute kidney injury score was calculated based on admission creatinine as previously described and examined as a dichotomous variable (no AKI = “0,” AKIN stage 1–3 = “1–3”).^18^

For practice-level data, quasi-Poisson regression modelling was used to manage over dispersion within the dataset.^19^ For patient-level data, mixed model binary logistic regression was performed with hospital admission as the dependent variable and hospital included as a random effect. Model assumptions were checked visually including colinearity between dependent variables and were deemed acceptable. Practice referral rate for acute medical assessment was expressed as referrals per 100 patient-years and entered as a continuous dependent variable with age, deprivation, care home residence, distance from hospital site and practice list size as explanatory variables. When interpreting the model output a *P* value of <0.05 was deemed significant. Model performance was assessed using observed versus expected counts with adjusted *R*^2^ to express goodness of fit. Patient-level analysis and graphical outputs of the referred population were performed (*n* = 42,424) using “finalfit” package.^20^ It is recognized that the presence of absence of an acute kidney injury is not available to primary care clinicians on initial assessment although clinical features associated with sepsis or dehydration may be present. As such AKIN score was not included in the reported model but sensitivity analysis was performed (Supplementary Table 1) demonstrating inclusion had minimal effect on statistical outcome. The study received local Caldicott Guardian approval and was undertaken in line with local information governance procedures. Analyses were performed using R versions 4.1.2 “Bird Hippie.”^21^ R script of full analysis can be found at https://github.com/marcus-lyall/GP-referral-variance.git.

## Results

**Fig. 2.**
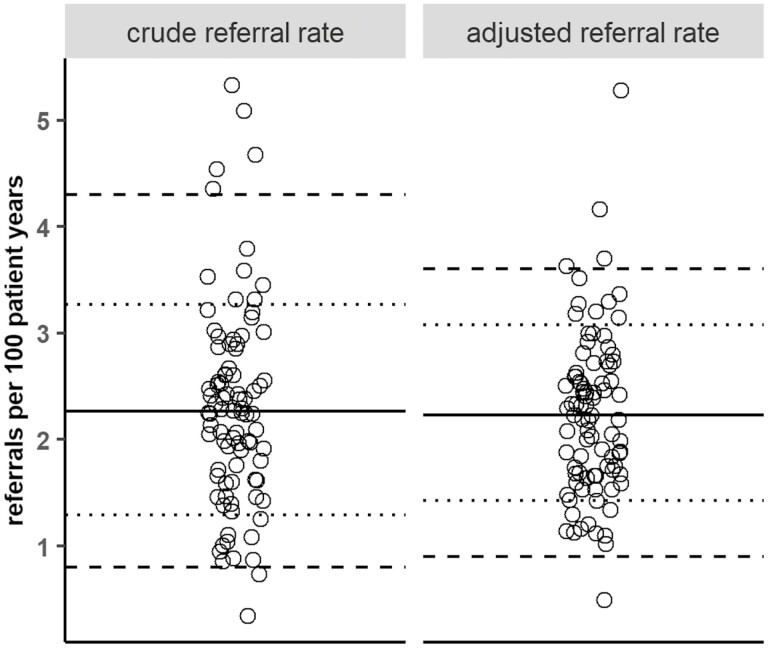
Crude and adjusted referral rates to acute medicine in Lothian, Scotland presented as referrals per 100 patient-years. Rate adjusted by quasi-Poisson regression. *n* = 42,424. Each dot = 1 GP. Solid horizontal line = median, dotted lines = median 1st and 4th quartiles, dashed lines = median 1st and 10th deciles.

There were 42,424 referrals for acute medical assessment from 88 practices during the study period. The median crude rate of referral per practice was 2.26 per 100 patient-years (min = 0.34, max = 5.33). This was equivalent to a 15.7-fold variation between the lowest and highest referring practice, a 4.9-fold variation between the median of the first and tenth deciles of referring practices (median first decile = 0.88, tenth decile = 4.36 referrals per 100 patient-years per practice) and a 2.53-fold variation between first and fourth quartiles (first quartile = 1.29, fourth quartile = 3.27 referrals per 100 patient-years) (Fig. 2).

Practice population age and deprivation were significantly associated with acute medical referral rate (*t* statistic 5.26 and 5.11, *P* < 0.01). There was a trend to negative association with care home residency (*t* statistic −1.98, *P* = 0.051) but there was no significant association with distance to hospital or practice list size (Table 1). The completed model explained 67% of the variation seen (observed/expected adjusted *R*^2^ 0.67) (Supplementary Fig. 1). Consistent with this, adjustment for confounders reduced but did not remove substantial variation in referral rate which remained 2.15-fold (1.43 and 3.07 referrals per 100 patient-years per practice between first and fourth quartiles) (Fig. 2).

**Table 1.** GP-level analysis of 42,424 referrals for acute medical assessment in Lothian, Scotland 2017–2020.

Variable	Median (IQR)	Estimate	Std error	*t* statistic	*P*
% population in bottom 2 SIMD quintiles	21.04 (28.13)	0.009	0.002	5.260	<0.001
% population in over 65	16.6 (8.06)	0.041	0.008	5.111	<0.001
% population over 65 in care home	1.11 (5.01)	−0.021	0.011	−1.982	0.051
distance to hospital (10 km)	0.43 (0.41)	−0.061	0.056	−1.096	0.276
Practice list size (1,000 patients)	7.62 (3.96)	−0.006	0.012	−0.499	0.619

Analysis performed by quasi-Poisson regression modelling for referral count against practice population with practice-level explanatory variables.

**Fig. 3.**
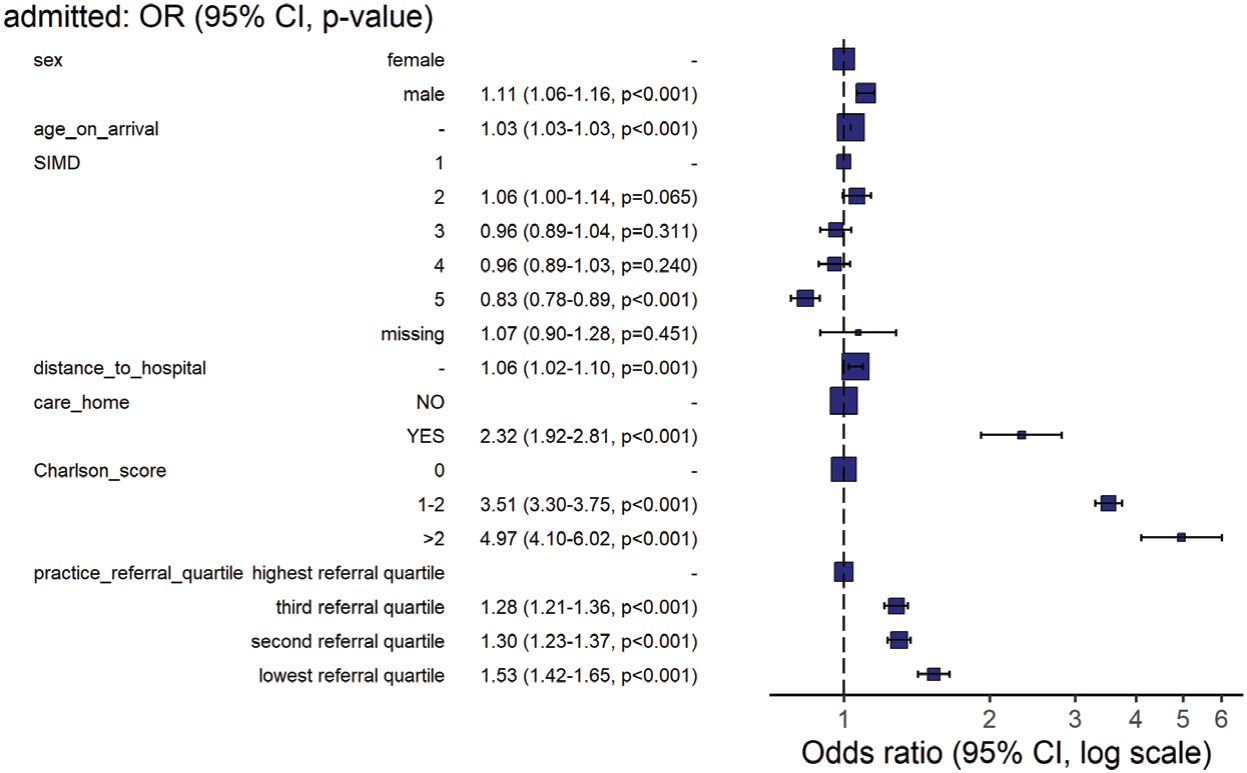
Forest plot of variables associated with requirement for acute medical admission following GP referral in Lothian, Scotland. Adjusted odd ratio by mixed model binary logistic regression with hospital site as random effect. Box width reflects patient number, error bars = 95% CI. *n* = 42,424.

Examining patient-level data for referred and clinically assessed patients demonstrated that increasing age, being out with the most affluent deprivation quintile and increasing Charlson comorbidity score were independently associated with need for medical admission (Table 2, Fig. 3, Supplementary Table1). Care home residency demonstrated an over 2-fold risk of admission with only 130/993 (13%) of patients referred from a care home being discharged following initial assessment (odds ratio [OR] 2.32 [1.92–2.81, *P* < 0.001]).

**Table 2.** Analysis of 42,424 referrals for acute medical assessment in Lothian, Scotland 2017–2020 and need for subsequent hospital admission.

Dependant variable = admitted to hospital following assessment	Immediate discharge (*n*[%])	Admitted to hospital (*n*[%])	OR (multilevel)
Sex			
Female	10,483 (59.8)	14,218 (57.1)	—
Male	7,039 (40.2)	10,684 (42.9)	1.11 (1.06–1.16, *P* < 0.001)
Age on arrival			
Mean (SD)	55.5 (19.4)	69.2 (18.0)	1.03 (1.03–1.03, *P* < 0.001)
SIMD quintile			
1	3,451 (19.7)	4,487 (18.0)	—
2	3,918 (22.4)	5,980 (24.0)	1.06 (1.00–1.14, *P* = 0.065)
3	2,740 (15.6)	3,873 (15.6)	0.96 (0.89–1.04, *P* = 0.311)
4	2,634 (15.0)	3,858 (15.5)	0.96 (0.89–1.03, *P* = 0.240)
5	4,467 (25.5)	6,372 (25.6)	0.83 (0.78–0.89, *P* < 0.001)
Missing	312 (1.8)	332 (1.3)	1.07 (0.90–1.28, *P* = 0.451)
Distance to hospital (10 km)			
Mean (SD)	0.6 (0.6)	0.7 (0.7)	1.06 (1.02–1.10, *P* = 0.001)
Care home residence			
No	17,392 (99.3)	24,039 (96.5)	—
Yes	130 (0.7)	863 (3.5)	2.32 (1.92–2.81, *P* < 0.001)
Charlson score			
0	15,982 (91.2)	17,013 (68.3)	—
0–2	1,416 (8.1)	7,015 (28.2)	3.51 (3.30–3.75, *P* < 0.001)
>2	124 (0.7)	874 (3.5)	4.97 (4.10–6.02, *P* < 0.001)
Practice referral quartile			
Highest referral quartile	7,496 (42.8)	9,071 (36.4)	—
Third referral quartile	3,998 (22.8)	5,936 (23.8)	1.28 (1.21–1.36, *P* < 0.001)
Second referral quartile	4,321 (24.7)	6,943 (27.9)	1.30 (1.23–1.37, *P* < 0.001)
Lowest referral quartile	1,707 (9.7)	2,952 (11.9)	1.53 (1.42–1.65, *P* < 0.001)

OR and *P* values calculated using mixed model binary logistic regression (*n* = 42,424) with hospital site included as random effect.

Patients from the highest referring quartile of GP practices were over 50% less likely to require hospital admission than those referred by the lowest referring practice quartile (OR 1.53 [1.41–1.66, *P* < 0.001]) and ~30% less likely to require admission than patients from practices within the interquartile range (third referral quartile = OR 1.28 [1.21–1.36, *P* < 0.01], second referral quartile = OR 1.30 [1.23–1.37, *P* < 0.001]). AKIN score, while an important marker of clinical acuity, is not available to primary care practitioners at the time of referral decision-making and was therefore included in a secondary sensitivity analysis. AKIN stages 1–3 were associated with increased likelihood of admission (adjusted OR 3.77 [3.45–4.13, *P* < 0.001]) but did not substantially change the effect of source practice referral quartile (Supplementary Table 1).

## Discussion

This large retrospective observational study demonstrates a high variation in referral rates between primary care GPs in Lothian for acute medical assessment which is incompletely explained by the available demographic variables of the practice populations studied. To our knowledge this is the first study to examine in detail GP referral practice for acute medical assessment.

At the practice level, referral variance was explained to a significant degree by the reported model (*R*^2^ 0.67) however significant variation persists after correction for established factors of high clinical need. It has been well described that age and deprivation are key correlates of emergency secondary healthcare access and so the finding that correction for these factors partially attenuates the degree of variation is expected.^11,12,22–24^ There was no significant effect of distance to hospital where a negative association may be expected. This variable was examined in a continuous fashion with the median distance or only 4.3 km (IQR 0.48). It may be that an effect does exist with rural (30–40 km) versus urban practices however this may be confounded by differences in rural practice organization. The finding that considerable variation continues to exist after correction for these variables is likely in part due to the lack of patient-level population wide data allowing detailed adjustment for deprivation, age, and comorbidity on an individual basis but perhaps also due to the influence of unmeasurable primary and secondary care factors identified in the conceptual framework (Fig. 1).

Interestingly, practice list size was not associated with an increase in referral rate. We hypothesized that larger practice list sizes would result in less individual patient care continuity, recently demonstrated to increase unscheduled care utilization, hospital admission, and mortality in the Norwegian population.^25^ It may be that increasing practice list size is not significantly associated with a change in care continuity or alternatively that reductions in continuity result only in an increase in patient emergency care self-presentation, not measured in these analyses.

There was a highly significant sequential negative association between the referral rate of the source GP and the likelihood for admission after adjustment for confounders. It is accepted that variation in admitting behaviour will exist between secondary care clinicians however approximately 30 individual senior physicians overseeing >100 individual junior clinicians assessed the referred patient populations at both hospital sites and as such there is no rationale this would bias 1 practice over another throughout the study period. Individual hospital site may influence referral rate due to hospital infrastructure referral pathways and ambulatory care availability and this was adjusted for in the mixed model methodology.

Detailed patient-level data were available for patients attending for acute medical assessment. These data demonstrate an expected increase in need for hospital admission with age, deprivation, comorbidity, care home residency, and the presence of an acute kidney injury. While age, comorbidity, and acute kidney injury are likely to reflect acuity of presenting pathology, the independent association between deprivation and need for admission is consistent with previous works and may also indicate reduced levels of available social support hindering early discharge.^24,26^ Interestingly, our analysis demonstrates a 2.3-fold independent association with care home residence and need for admission after medical assessment with only 13% of care home residents immediately discharged. The authors suggest a major barrier to early discharge in these patients is the need for ambulance transport which is not available until to the following day. Patients in the care home setting already have domiciliary support in place and in many cases may be better served community hospital at home services. Widening access to and improving utilization of these services as well as ensuring appropriate individual care plans provides an opportunity to reduce referrals and improve patient experience in the care home population.

Taken together these findings suggest that variation in primary care clinician risk threshold, patient population preference or expectation and perhaps GP culture plays an important role in the adjusted variance observed. Previous studies have examined outpatient referral and also total medical admission rates from all sources.^7,8,11,12^ However, these studies were not specific to the acute medicine/primary care interface, which is under extreme pressure in the UK health service. O’Donnell et al. reviewed the literature up to the year 2000 and concluded that the measured patient demographics, individual practice, and individual general practitioner factors were responsible for only a minority of the observed variance in referrals with the majority of variance unexplained.^7^ This review extensively focussed on nonurgent referrals for outpatient assessment with a minor focus on acute assessment and only 8 of 29 studies included inpatient referrals. Further these included referrals for all specialties including surgery, mental health, and paediatrics and the majority of included data are now over 20 years old and may not accurately reflect current practice in the rapidly changing health care landscape. A more recent analysis of ~130,000 patient referrals from the health improvement network database in primary care demonstrated that age, gender, and deprivation significantly affected referral behaviour in a disease-specific manner. Referring clinician gender and experience factors may also be important^9,27^ and targeted education with rolling feedback to general practitioners may significantly reduce variation in practice.^10^ High variation in referral rates to the emergency department and direct specialty admission at the clinician level in the out of hours GP setting as also been reported, which may be due to differences in clinical trainee status, locality of in hours work or by GP attitudes and beliefs around referral threshold.^28–30^

Strengths of this study include its size and duration of study period. GP personnel fluctuate with periods of leave and locum use, which could affect referral patterns. The examination of a 2 year 9 month period should have ameliorated the impact of these factors. The use of a single computer-based clinical management systems across the region allowed a highly specific dataset to be curated for patients referred for medical assessment by their GP practice with the exclusion of self-presenting patients, out of hours GP referrals, ambulance service referrals and those patients immediately triaged to minor injury, surgical and other specialty services. In addition, the practice where the patient was currently registered was recorded on referral, compensating for those moving practice during the study.

A key limitation in this study is the use of group practice as an independent variable and the population-level explanatory variables assigned to each practice to explain variation. Analysis at the practice-level measures the collective referral behaviour of up to twenty general practitioners which may vary widely and reflect not only the referral practice of the clinician but the organizational infrastructure of the practice. Clinician-level factors such as experience, skill mix, and gender known to be associated with differences in referral rate are unaccounted for in this study.^9,27,29–31^ Practice-level factors such as access to appointments, training practice status, the presence and utilization of extended evening or weekend hours service and reliance on locum staff may also have significant effect and cannot be controlled for in this study.^31^ Community factors such as hospital at home access, community hospital access, crime rates, and transport links may play an important role and data to adjust for these are lacking. In addition, individual patient-level factors such as prior healthcare contact, prescribing burden, educational level, marital status, and household support may also play a key role not adequately represented in the SIMD variable. Increasing granularity and linkage of routinely collected healthcare and national disease databases coupled with advances machine learning to assign relative weight to these factors shows promise at predicting hospital admission and may help clarify key dependent variables in future.^32^

We have used need for medical admission as a measure of GP practice or patient population risk tolerance and, in essence, assumed that practices where a high proportion of patients were immediately discharged, demonstrated a lower tolerance for management in the community setting. It is important to note that need for admission is not an absolute indicator of an appropriate referral. Patients with complex home support are often admitted due to transport issues and departmental crowding when they may not require inpatient management. Furthermore, referral for exclusion of potentially serious pathology such as myocardial infarction, pulmonary embolus, and subarachnoid haemorrhage, following which a patient is discharged, is often entirely appropriate. The optimal admission to discharge ratio for a given population is currently unknown.

Further work is now required to identify the population of patients referred to acute medicine who could conceivably have been managed in the community and to identify modifiable community and GP-level factors associated with high acute medicine utilization. Populations for whom acute medical referral could be avoided are patients with a low risk of pathology for whom outpatient investigation or rapid access clinic (chest pain, respiratory, neurovascular, ambulatory care) would suffice and patients with a high level of frailty or physical dependence for whom urgent social support is not available. Future research in this field must include more detailed patient- and consultation-level data to examine both likelihood of referral for specific complaint and subsequent clinical outcome. Point of care testing, strengthening primary and secondary care communication with high referring practices and extending ambulatory care services and alternatives to acute medicine referral may all play a role in reducing variation in referral rate and relieving highly congested acute unscheduled care pathways.^10,33–36^

## Conclusions

There is a high variation in referral rates for acute medical assessment between GPs in this study, a significant proportion of which may be due to practice level, patient or community factors that are as yet unexplained. When this is achieved there may be an opportunity to optimize the use of acute medical assessment units and advance the current strategic vision of community-based care.

## Supplementary Material

cmac097_suppl_Supplementary_Material

cmac097_suppl_Supplementary_Checklist

## Data Availability

The component datasets used here are available via the Public Benefits Privacy Panel for Health at https://www.informationgovernance.scot.nhs.uk/pbpphsc/ for researchers who meet the criteria for access to confidential data. All source code use for variable derivation, analysis, and plot generation is available at https://github.com/marcus-lyall/GP-referral-variance.git.
