# Supplementary material for: Variation in general practice referral rate to acute medicine services and association with hospital admission. A retrospective observational study
Source: Fam Pract. 2022 Sep 5;40(2):233–40. doi: 10.1093/fampra/cmac097 (PMC10047615; doi:10.1093/fampra/cmac097)
Supplement: cmac097_suppl_Supplementary_Material [file cmac097_suppl_supplementary_material.docx]

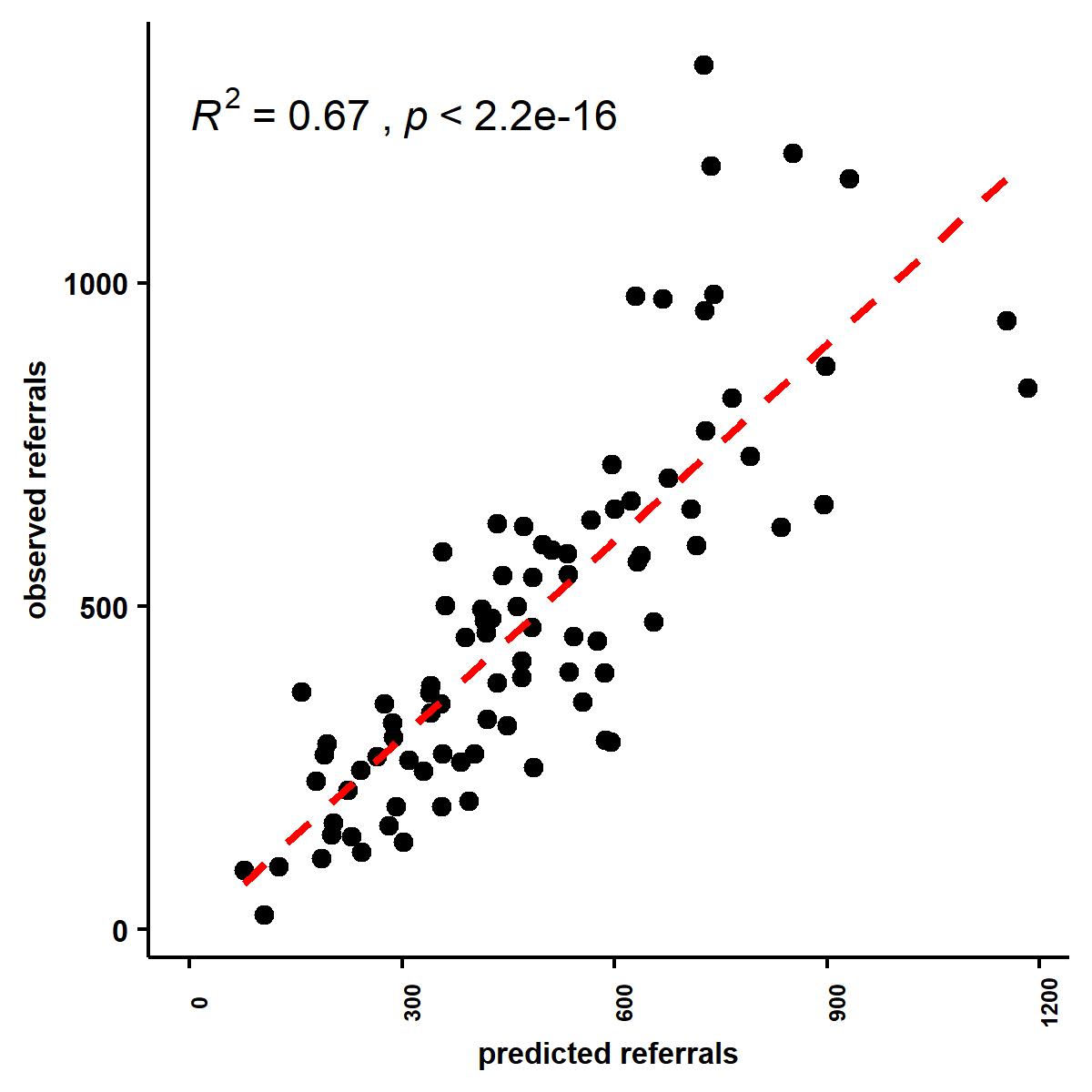


Supplementary Figure 1: quasi-Poisson model performance of GP referral counts utilising age, deprivation, care home residence, distance to hospital and practice list size as explanatory variables. R^2^ and p value calculated by Pearson correlation co-efficient.

Supplementary table 1

Mixed model binary regression output of likelihood of immediate discharge following GP referral for acute medical assessment against explanatory variables with hospital site as a random effect.

| Dependant variable = Hospital admission following assessment. |  | immediate discharge n (%) | admitted to hospital n (%) | Adjusted Odds Ratio |
| --- | --- | --- | --- | --- |
| SEX | female | 10483 (59.8) | 14218 (57.1) | - |
|  | male | 7039 (40.2) | 10684 (42.9) | 1.04 (0.99-1.08, p=0.117) |
| Age on arrival | Mean (SD) | 55.5 (19.4) | 69.2 (18.0) | 1.03 (1.03-1.03, p<0.001) |
| SIMD quintile | 1 | 3451 (19.7) | 4487 (18.0) | - |
|  | 2 | 3918 (22.4) | 5980 (24.0) | 1.07 (1.00-1.14, p=0.052) |
|  | 3 | 2740 (15.6) | 3873 (15.6) | 0.97 (0.90-1.04, p=0.365) |
|  | 4 | 2634 (15.0) | 3858 (15.5) | 0.97 (0.90-1.05, p=0.425) |
|  | 5 | 4467 (25.5) | 6372 (25.6) | 0.85 (0.79-0.91, p<0.001) |
|  | missing | 312 (1.8) | 332 (1.3) | 1.08 (0.90-1.29, p=0.426) |
| Distance to hospital (10km) | Mean (SD) | 0.6 (0.6) | 0.7 (0.7) | 1.06 (1.02-1.10, p=0.001) |
| Care home residence | no | 17392 (99.3) | 24039 (96.5) | - |
|  | yes | 130 (0.7) | 863 (3.5) | 2.33 (1.92-2.82, p<0.001) |
| Charlson score | 0 | 15982 (91.2) | 17013 (68.3) | - |
|  | 2-Jan | 1416 (8.1) | 7015 (28.2) | 3.48 (3.27-3.71, p<0.001) |
|  | >2 | 124 (0.7) | 874 (3.5) | 4.81 (3.96-5.84, p<0.001) |
| AKIN score group | AKIN 0 | 16503 (94.2) | 20772 (83.4) | - |
|  | AKIN 1-3 | 656 (3.7) | 4025 (16.2) | 3.77 (3.45-4.13, p<0.001) |
|  | missing | 363 (2.1) | 105 (0.4) | 0.25 (0.20-0.32, p<0.001) |
| Practice referral quartile | highest referral quartile | 7496 (42.8) | 9071 (36.4) | - |
|  | third referral quartile | 3998 (22.8) | 5936 (23.8) | 1.26 (1.19-1.33, p<0.001) |
|  | second referral quartile | 4321 (24.7) | 6943 (27.9) | 1.30 (1.23-1.38, p<0.001) |
|  | lowest referral quartile | 1707 (9.7) | 2952 (11.9) | 1.52 (1.41-1.64, p<0.001) |

OR = adjusted odds ratio. OR and p values calculated using mixed model binary logistic regression (n = 42424) with hospital site included as random effect. AKIN = Acute Kidney Injury Network score.
